# Subtle diastolic dysfunction in children with idiopathic subclinical hypothyroidism: effect of two years of l-thyroxine treatment

**DOI:** 10.1007/s40618-026-03002-2

**Published:** 2026-07-30

**Authors:** M. Arcopinto, M. Salerno, R. Di Mase, A. Salzano, F. R. Rotondo, S. Vasaturo, A. Cittadini, D. Capalbo

**Affiliations:** 1 Department of Translational Medical Sciences, University of Naples Federico II, Naples, Italy; 2 Interdepartmental Center for Gender Medicine Research “GENESIS”, University of Naples Federico II, Naples, Italy; 3 Pediatric Endocrinology Unit, Department of Mother and Child, University Hospital Federico II of Naples, Endo-ERN Center for Rare Endocrine Conditions, Naples, Italy; 4 Pediatric Endocrinology Unit, Department of Translational Medical Sciences, University Hospital Federico II of Naples, Naples, Italy

**Keywords:** Subclinical hypothyroidism, Children, Heart, Diastolic function, Levothyroxine

## Abstract

**Purpose:**

Data about clinical implications of subclinical hypothyroidism (SH) in children are scanty, and the management of this condition is still challenging particularly for mild forms (TSH levels ranging between 4.5 and 10 mU/L). Aim of this study is to compare left ventricular (LV) geometry and function in SH children and matched euthyroid controls, and to evaluate the cardiac effects of L-thyroxine (L-T_4_) therapy.

**Methods:**

Thirty-six children (19 females), aged 8.3 ± 0.7 years, with persistent and idiopathic SH were enrolled in the study. Thirty-six euthyroid children comparable for sex, age and body mass index were enrolled as controls. Baseline echocardiography and biochemical assessment was performed in all subjects. Thereafter, SH children started L-T_4_ therapy. Both assessments were repeated after 2 years of therapy in patients and 2 year -follow-up in controls.

**Results:**

The LV cavity size was comparable between SH and controls, at baseline, and increased similarly over time. LV systolic function was normal at baseline in both groups and remained comparable at end-study. With regard to diastolic function, SH children showed a significant prolongation of isovolumic relaxation time (IVRT) compared with controls (92.95 ± 3.74 vs 76.13 ± 2.75 msec, p = 0.0006), with normal E/A and E/E’ ratios in both populations, consistent with subclinical, mild diastolic dysfunction. After L-T_4_ therapy, IVRT significantly decreased (81.43 ± 4.28 msec *p* < 0.05) becoming similar to controls (82.20 ± 2.14 msec).

**Conclusion:**

Long-lasting mild SH in children is associated with subtle abnormality of LV relaxation which improves with L-T_4_ therapy. Whether these subtle alterations may lead to significant clinical consequences require further investigation.

## Introduction

The relationship between thyroid hormone and the cardiovascular (CV) system has been extensively documented [1–4]. The heart is one of the major target organs for thyroid hormone action, and changes in cardiac function may occur in patients with both overt and subclinical thyroid failure [2–3, 5]. CV abnormalities in adults with thyroid hormone deficiency are mainly characterized by left ventricular (LV) diastolic dysfunction [6], reduced systolic function, systolic and diastolic dysfunction during exertion, and increased systemic vascular resistance [5, 7].

Mild CV abnormalities have been also documented in subjects with subclinical hypothyroidism (SH). SH is a biochemical condition characterized by elevated thyrotropin (TSH) levels while free thyroxine (FT4) concentrations remain within the normal range. Based on the degree of TSH increase, SH can be classified as mild—when TSH levels fall between the upper limit of the reference range and 9.99 mIU/L—or severe, when TSH is or exceeds 10 mIU/L [8–9]. LV diastolic dysfunction is the most common cardiac alteration associated with SH in adulthood [10–11]. Furthermore, LV systolic function at rest may also be mildly impaired, as indicated by a prolonged pre-ejection period, an increased pre-ejection period/LV ejection time ratio and a reduced mean aortic acceleration in SH versus euthyroid controls [12–14]. Noteworhty, several studies and meta‑analyses have reported an association between SH and an increased risk of coronary events and CV mortality in adults, a risk that appears particularly evident in younger individuals and in those with TSH levels > 10 mU/L [6, 15–18]. Although some authors documented a beneficial effect of LT4 therapy, the efficacy of treatment in improving cardiac dysfunction in adults with SH is still debated [4, 19, 20].

The evidence on the overall management and CV implications of SH in children is still limited [8, 21–22]. Some authors have suggested that mild, long‑standing untreated SH in children may be associated with a cluster of early metabolic risk factors for CV disease [23–25]; these alterations appear to improve after treatment with LT4 [26–27]. Conversely, data on systolic and diastolic function are still very scanty.

Aim of this prospective case-control study was to assess the LV morphology and function in children with persistent mild idiopathic SH compared with euthyroid controls, and to evaluate the effects of a two-year L-T_4_ replacement therapy on the CV system in SH subjects.

## Subjects and methods

### Subjects

Thirty-six children (19 females and 17 males) aged 8.3 ± 0.7 years, with mild, persistent and idiopathic SH were enrolled in the study. The mean duration of SH was 3.7 ± 0.5 years.

Inclusion criteria were: (1) TSH levels between 5.0 and 10 mU/L with FT4 values within the reference range, persisting for at least 2 years prior to enrollment; (2) absence of anti-thyroglobulin (Tg-Ab) and anti-thyroperoxidase (TPO-Ab) antibodies, and normal thyroid echogenicity on ultrasound; (3) absence of palpable goiter, symptoms of hypothyroidism, chromosomal abnormalities, or chronic diseases; (4) adequate urinary iodine excretion.

Thirty‑six healthy euthyroid children, comparable in sex, age, body mass index (BMI), and pubertal status, were included as controls. They were selected from a cohort of healthy subjects recruited through a cooperative study program between our center and local family pediatricians.

All subjects were prepubertal at baseline; 9 SH children and 9 controls entered puberty during the study.

The study protocol was approved by the Ethics Committee of the Federico II University of Naples and informed consent was obtained from all parents.

### Study protocol

At study entry both SH subjects and controls underwent a complete clinical evaluation including measures of height, weight, and heart rate. BMI was calculated as weight (kilograms)/height (meters)^2^. Height and BMI were normalized by age and sex according to Italian standards and expressed as standard deviation score (SDS) [28]. Systolic and diastolic blood pressure (SBP, DBP) were measured at the right arm by a cuff manometer after 5 min of supine rest according to current guidelines [29].

TSH, FT_4,_ Tg-Ab and TPO-Ab were assessed in both SH and controls at 8 a.m. after an overnight fast and thyroid ultrasound was performed as a part of baseline evaluation.

Both populations underwent a complete Doppler echocardiography at study entry.

After the basal evaluations, the SH subjects started a two-year L-T_4_ treatment. The initial dose of 50 µg/day (corresponding to 1–2 mcg/kg/day) for all SH subjects was then adjusted according to hormonal response to maintain TSH and FT_4_ serum levels within the reference ranges.

Clinical, hormonal and echocardiographic parameters were re-assessed in both SH subjects, after a period of two years of L-T_4_ treatment, and in controls after a 2-year observation.

### Echocardiography

An ultrasound system equipped with a 2.5 MHz multi-frequency transducer (Aplio, Toshiba, Japan) was used for complete M-mode, two-dimensional, Doppler and Tissue Doppler Imaging (TDI) echocardiographic analyses. M-mode and two-dimensional recordings were made with the patients in the lateral recumbent position, according to previously published methods [30]. Measures of LV morphology and function were assessed according to standard formulae. Relative Wall Thickness (RWT), a measure of LV remodeling, was calculated as $\:2\times\:\frac{Posterior\:wall\:thickness}{End\:diastolic\:diameter}$. Measures of LV end-diastolic volume (EDV) and end-systolic volume (ESV) were measured by the modified Simpson’s rule. Accordingly, LV ejection fraction (EF) was calculated as follows:$\:EF=\frac{\left(\mathrm{E}\mathrm{D}\mathrm{V}-\mathrm{E}\mathrm{S}\mathrm{V}\right)}{EDV}\:100$. LV mass was calculated according with American Society of Echocardiography-recommended formula: $LV\:mass=0.8\:\times\:\{1.04 [{(LVIDd+PWTd+SWTd)}^{3}$ $-{\left(LVIDd\right)}^{3}]\} +0.6\:\mathrm{g}$ where PWTd and SWTd are posterior wall thickness at end diastole and septal wall thickness at end diastole, respectively [31]. The following parameters of diastolic function were measured as the mean of three to five consecutive beats: diastolic transmitral peak velocities ratio (*E/A* ratio), Isovolumic Relaxation Time (the time interval between the aortic closure and the mitral opening, IVRT), Mitral deceleration Time (time interval from the peak velocity of passive transmitral flow during LV filling and diastasis, MdT) and pulmonary vein velocities. Quantitative diastolic data were derived from Tissue Doppler (TDI) analysis placing the sample volume (4 mm^3^) in the LV basal portions of the septal and lateral walls. To estimate the LV filling pressure, the ratio of transmitral Doppler early filling velocity to tissue Doppler early diastolic mitral annular velocity was calculated ($E/E^{\prime}$ ratio) [32], . Height was used for indexing LV mass, LV end-systolic volume and LV end-diastolic volume. Measurement of all parameters was performed off-line by one experienced investigator blind to the study protocol (M.A.)

### Statistical analysis

All variables were tested for normal distribution using the Kolmogorov-Smirnov test. Skewed variables (HR, PAS, indexed LVEDV and LVESV) are expressed as median (interquartile range). Continuous variables are expressed as means ± standard error of the mean (SEM). Normally distributed variables were compared between two groups using the two-sided, unpaired Student’s *t*-test with the assumption of unequal variance. Mann-Whitney test was used to compare variables with skewed distribution. T test for paired samples was used to compare baseline and end-study observations in both SH subjects and controls. Significance was set at 5%. Statistical analysis was performed using the SPSS version 19.0 package (SPSS Inc., Chicago).

Differences between groups were evaluated by linear models (ANCOVA). Differences in changes from baseline over the two years of the study in SH patients compared to healthy controls were evaluated through linear model entering basal values of each parameter as covariates (ANCOVA). Bivariate correlation was used to evaluate the relationship between variables.

## Results

Clinical, hormonal, and echocardiographic features of SH subjects and controls at baseline and after the 2‑year follow‑up are summarized in Table 1. SH subjects had similar anthropometric characteristics compared with controls being the two populations matched for age, sex, BMI. There was no difference in resting systolic or diastolic blood pressure as well as heart rate between patients and controls.

As expected for SH subjects, serum TSH levels were significantly higher than in controls. FT4 mean values were within the normal range in both populations and were similar in SH and controls. LV cavity size was comparable between groups; indeed, no differences in wall thickness, relative wall thickness (RWT) and indexed LV mass were found in the study populations.

**Table 1:** Clinical, hormonal and echocardiographic assessment in controls and SH subjects at baseline and after 2-year L-T4 treatment in SH subjects or follow-up in controls

	Baseline	2 yr	*p* ^a^	*p* ^b^
*Age, (yrs)*			0.98	0.74
Controls (*n* = 36)	8.27 ± 0.73	10.25 ± 0.73		
SH (*n* = 36)	8.25 ± 0.88	10.62 ± 0.82		
*Height, SDS*			0.11	0.33
Controls	− 1.27 ± 0.27	− 1.11 ± 0.31		
SH	− 0.60 ± 0.32	− 0.71 ± 0.27		
*Body mass index, SDS*			0.59	0.21
Controls	− 0.12 ± 0.34	− 0.19 ± 0.22		
SH	0.12 ± 0.28	0.30 ± 0.32		
*TSH (mU/mL)*			< 0.001	0.07
Controls	2.50 ± 0.18	2.41 ± 0.22		
SH	6.13 ± 0.23	2.96 ± 0.21		
*FT*_4_ *(ng/dL)*			0.69	0.13
Controls	1.29 ± 0.03	1.28 ± 0.03		
SH	1.27 ± 0.04	1.37 ± 0.05		
*Systolic arterial pressure (mmHg)*			0.45	0.21
Controls	100.50 ± 1.88	107.16 ± 1.94		
SH	102.89 ± 2.56	112.65 ± 3.84		
*Diastolic arterial pressure (mmHg)*			0.81	0.42
Controls	61.67 ± 2.23	60.79 ± 2.01		
SH	60.88 ± 2.47	62.68 ± 1.16		
*Heart rate (bpm)*			0.35	0.24
Controls	84.47 ± 3.42	73.67 ± 1.66		
SH	88.38 ± 2.31	78.42 ± 3.65		
*Interventricular septum thickness, diastole (mm)*			0.64	0.91
Controls	5.79 ± 0.18	6.44 ± 0.19		
SH	5.94 ± 0.26	6.48 ± 0.28		
*Interventricular septum thickness, systole (mm)*			0.66	0.50
Controls	8.26 ± 0.38	9.03 ± 0.39		
SH	8.46 ± 0.24	9.43 ± 0.44		
*LV end-diastolic diameter (mm)*			0.83	0.51
Controls	37.59 ± 1.56	42.04 ± 1.33		
SH	38.01 ± 1.12	40.67 ± 1.53		
*LV end-systolic diameter (mm)*			0.90	0.20
Controls	24.76 ± 1.36	29.59 ± 1.37		
SH	24.97 ± 0.98	27.51 ± 0.84		
*Relative wall thickness*			0.48	0.48
Controls	0.30 ± 0.01	0.30 ± 0.03		
SH	0.31 ± 0.01	0.32 ± 0.01		
*LV mass, indexed* (g/m^2,7^-height)			0.67	0.34
Controls	31.09 ± 1.63	30.60 ± 2.58		
SH	30.36 ± 2.68	34.04 ± 2.72		
*LV end-diastolic volume, indexed* (ml/m^2,7^-height)			0.32	0.39
Controls	26.35 ± 2.38	24.82 ± 2.19		
SH	29.58 ± 2.25	27.14 ± 1.52		
*LV end-systolic volume, indexed* (ml/m^2,7^-height)			0.52	0.14
Controls	10.49 ± 1.01	10.18 ± 0.71		
SH	11.49 ± 1.19	9.10 ± 0.10		
*LVEF (%)*			0.67	0.59
Controls	63.04 ± 2.63	63.96 ± 1.45		
SH	61.73 ± 1.66	62.82 ± 1.49		
*LV ejection time (msec)*			0.44	0.22
Controls	268.73 ± 5.32	284.00 ± 4.15		
SH	262.65 ± 5.96	292.43 ± 5.47		
*IVRT (msec)*			0.0006	0.047
Controls	76.13 ± 2.75	82.20 ± 2.14		
SH	92.95 ± 3.74	81.43 ± 4.28		
*MdT (msec)*			0.57	0.60
Controls	146.56 ± 6.92	162.29 ± 7.77		
SH	141.83 ± 4.54	168.95 ± 9.83		
*E/A ratio*			0.91	0.09
Controls	1.99 ± 0.09	2.12 ± 0.15		
SH	1.96 ± 0.23	1.80 ± 0.11		
*E/E’ ratio*			0.64	0.29
Controls	6.82 ± 0.35	6.17 ± 0.52		
SH	6.62 ± 0.25	7.24 ± 0.87		

Data expressed as mean ± SEM. LV: left ventricle; IVRT: isovolumic relaxation time; MdT: mitral deceleration time; E/A ratio: ratio of diastolic trans-mitral peak velocities; E/E’ ratio: ratio of transmitral Doppler early filling velocity to tissue Doppler early diastolic mitral annular velocity

^a^SH subjects at baseline vs. controls; ^b^SH subjects after 2 years of L-T4 therapy vs. SH subjects at baseline

Systolic function, expressed as LVEF, was normal and comparable in SH and control populations (61.73 ± 1.66 vs. 63.04 ± 2.63, *p* = 0.67).

Regarding diastolic function, SH children showed a significant prolongation of IVRT compared to controls (92.95 ± 3.74 vs. 76.13 ± 2.75 msec, *p* < 0.0006), with normal *E/A* waves and $E/E^{\prime}$ ratios in both populations.

Mean changes of most measures of LV morphology and function over the 2 years, compared through ANCOVA analysis, are summarized in Table 2. As expected, TSH and FT_4_ values normalized in treated-SH (t-SH) subjects and became comparable to controls at the end of the study.

**Table 2:** Mean changes of measures of LV morphology and function from baseline over the 2 years of L-T4 therapy in patients with t-SH compared with controls (ANCOVA)

	SH mean [95% CI]	Controls mean [95% CI]	*p*
Systolic arterial pressure (mmHg)	3.06 [1.02, 10.71]	6.94 [1.96, 11.65]	0.78
Diastolic arterial pressure (mmHg)	1.88 [− 1.18, 4.94]	− 0.17 [− 3.15, 3.40]	0.54
Heart rate (bpm)	− 11.44 [− 16.37, − 7.83]	− 10.78 [− 14.88, − 2.72]	0.37
Interventricular septum thickness, diastole (mm)	0.54 [1.12, 2.06]	0.66 [0.4, 1.14]	0.07
LV end-diastolic diameter (mm)	2.67 [0.28, 5.21]	4.45 [1.48, 7.22]	0.39
LV end-systolic diameter (mm)	2.54 [0.76, 4.46]	4.83 [3.34, 7.81]	0.12
Relative wall thickness	0.00 [0.01, − 0.10]	0.01 [− 0.07, 0.05]	0.81
LV mass, indexed (g/m^2,7^-height)	3.68 [2.81, 12.34]	− 0.48 [− 5.81, 5.29]	0.19
LV end-diastolic volume, indexed (ml/m^2,7^-height)	− 1.78 [− 4.25, 0.85]	− 1.82 [− 4.44, 0.65]	0.91
LV end-systolic volume, indexed (ml/m^2,7^-height)	− 0.73 [− 2.65, 1.84]	− 0.76 [− 3.24, 1.11]	0.67
LVEF (%)	1.10 [− 2.19, 4.55]	1.06 [− 1.88, 3.90]	0.93
LV ejection time (msec)	26.36 [15.49, 36.95]	17.88 [6.07, 33.45]	0.45
IVRT (msec)	− 11.52 [− 17.48, − 0.57]	6.07 [− 5.52, 10.78]	0.004
MdT (msec)	34.58 [10.54, 47.18]	18.12 [0.30, 37.99]	0.46
E/A ratio	− 0.16 [− 0.43, 0.72]	0.23 [− 0.15, 0.40]	0.10
E/E’ ratio	0.91 [− 0.90, 3.01]	0.26 [− 2.32, 0.67]	0.12

t-SH: treated SH; LV: left ventricle; IVRT: isovolumic relaxation time; MdT: mitral deceleration time; E/A ratio: ratio of diastolic trans-mitral peak velocities; E/E’ ratio: ratio of transmitral Doppler early filling velocity to tissue Doppler early diastolic mitral annular velocity

LV chamber diameters, volumes, wall thickness and indexed mass increased similarly both in t-SH and in controls, remaining within normal ranges for age.

LV diastolic function significantly changed in SH patients after L-T4 treatment; indeed, IVRT significantly decreased in t-SH (*p* = 0.004) and reached values comparable to those of the control group after 2 years of follow-up. The pattern of transmitralic flow ($$\:\raisebox{1ex}{$E$}\!\left/\:\!\raisebox{-1ex}{$A$}\right.$$ ratio), and the estimation of LV filling pressure with $$E/E^{\prime}$$ ratio did not change at the end of follow-up both in t-SH and in controls (Table 2).

Regarding systolic function, no significant differences were observed between SH and controls in LVEF and LV ejection time.

## Discussion

In the present study, we provide evidence that SH in children is associated with subtle abnormalities in LV diastolic function, while LV systolic function and geometry are not impaired.

Mild SH in children is generally a benign and remitting condition with a low risk of progression to overt hypothyroidism, particularly in non-autoimmune forms [8, 33–36]. However, data on long-term outcome in these children remain limited.

Height, growth velocity, and neurocognitive function have generally been reported as normal in children with mild non-autoimmune SH [35, 37–42].

However, a few studies have reported subtle pro‑atherogenic abnormalities in children with mildly elevated TSH levels [23–25, 43, 44], with L‑T4 replacement therapy exerting a beneficial effect on these alterations [26, 27]. Data on cardiac function in the pediatric population with SH are scanty. To better address this point, we designed this prospective, case-control study in children with long-lasting, mild, idiopathic SH to evaluate ventricular morphology and function before and after L-T4 treatment.

We documented that IVRT, defined as the time interval between aortic valve closure and mitral valve opening, was significantly higher (+ 26%) in children with SH compared with controls, although values remained within the normal range. Other markers of diastolic function, including MdT, *E/A* ratio and $E/E^{\prime}$ ratio were within normal limits and similar between the two study groups. Overall, this pattern is consistent with an isolated, mild, preclinical impairment of the relaxation phase of the cardiac cycle, without evidence of increased LV diastolic filling pressure.

The pathophysiologic correlation between a hypothyroid condition, although mild, and impaired diastole is not a novel finding: the relaxation process is an energy-dependent phase of the LV filling, characterized by calcium re-uptake toward the sarcoplasmic reticulum [45]. Thyroid hormones regulate the expression of several key proteins involved in intracellular calcium handling and cardiac contractility, including sarcoplasmic reticulum Ca²⁺-ATPase (SERCA2a), phospholamban, and myosin heavy chain isoforms. Impaired thyroid hormone signaling has been already associated with reduced SERCA2a expression, altered calcium homeostasis, and diastolic dysfunction (2). Moreover, thyroid hormones homeostasis is critical in the energetic utilization and LV relaxation is sensitive to even mild energy depletion. Abnormal relaxation results in continuous pressure generation at end diastole with possible reduction in LV distensibility [46].

The precise cellular and molecular mechanisms by which thyroid exerts its action have been well worked out and imply the regulation of the expression of key structural and regulatory genes (i.e. myosin heavy chain, regulators of intracellular calcium handling), responsible for contractile function and diastolic relaxation in the heart [2].

Cardiovascular abnormalities have been well documented in adults with mild SH [9]. In particular, in accordance with our data in children, SH has been associated with a higher prevalence of LV diastolic dysfunction compared with euthyroid individuals [15, 47]; this abnormality is characterized by an impaired LV relaxation pattern, generally evidenced by prolonged deceleration time, prolonged isovolumic relaxation time, and higher LV filling pressure [6, 14, 48–51].

Whether L-T4 replacement therapy provides long-term beneficial effects on CV alterations remains controversial [4, 11, 19]. Nevertheless, several studies suggest that normalization of TSH levels with L‑T4 may improve cardiac dysfunction in adults with SH [12, 14, 52] and, consistently, a recent meta‑analysis including 294 patients demonstrated that L‑T4 was associated with a significant improvement in diastolic function, with no substantial changes in cardiac morphology [53]. Similarly, Liu et al. [11] reported that thyroid hormone replacement may partially ameliorate diastolic dysfunction, albeit without achieving complete normalization of diastolic parameters.

Experimental studies have demonstrated the expression of TSH receptors (TSHR) in cardiomyocytes and other cardiovascular cell types, suggesting that TSH may exert direct biological effects independent of thyroid hormone signalling [54]. Although the functional relevance of cardiac TSHR activation in humans remains incompletely understood, it is conceivable that the normalization of TSH may have directly contributed, at least in part, to the recovery of diastolic function, independently of significant thyroid hormones fluctuations. However, given the limited clinical evidence currently available, this mechanism should be regarded as hypothesis-generating rather than causal.

In this context, the results of the present study, provide valuable insights, showing that two years of L-T4 therapy are associated with a reduction in IVRT, thus suggesting a positive effect of LT4 replacement on left ventricular relaxation properties.

A previous study enrolling children with Down syndrome and SH did not find alterations in myocardial structure and function [55]; in contrast, another study [56] reported that obese children with SH exhibited mild diastolic dysfunction and impaired left ventricular longitudinal systolic function compared with euthyroid obese children. However, these findings are difficult to interpret in the context of our study, as obesity is itself a well-established and independent determinant of cardiac structure and function. Furthermore, that study did not evaluate the effects of either weight loss or levothyroxine treatment on cardiac function [56]. Conversely, our study population was specifically selected to investigate the direct effect of SH on cardiac function, therefore patients with relevant comorbidities were excluded to minimize potential confounding factors.

Accordingly, other pediatric, case-control studies consistently support the presence of early subclinical alterations in left ventricular diastolic function in children with SH in line with our findings, although most of such studies are cross-sectional in design and based on short-term follow-up. Irdem et al. [57] documented the presence of echocardiographic markers of diastolic dysfunction in 30 children with SH compared with age-matched controls; in this study, IVRT was increased, as in our SH population (72.2 vs. 63.3 msec, + 14%), and other conventional ultrasound signs of diastolic impairment, including mitral E-wave velocity and *E/A* ratio decrease and Mitral Deceleration time prolongation, were detected. In the study by Arslan et al. [58] the mean mitral IVRT value of the SH group was 57.2 ± 9.3 msec, compared to 44.5 ± 5.6 msec for the control group (*p* < 0.0001), in keeping with our finding in terms of absolute values (+ 28%). Interestingly, the tricuspid IVRT was also evaluated, and it was also found to be increased in the SH group. Consistently with our baseline observation, there were no significant differences in the other m-mode or pulse Doppler echocardiography values between two groups.

In the case–control prospective study by Catli et al. [59], cardiac function was evaluated in 31 children (mean age 10.3 years) with SH secondary to Hashimoto thyroiditis, thyroid hypoplasia, or idiopathic thyroid disease. Compared with healthy controls, children with SH exhibited increased LV wall thickness and prolonged IVRT (+ 14%), whereas indexed LV diameters were comparable between the two groups. Consistent with our findings, IVRT significantly decreased to values comparable to controls following L-T4 therapy, supporting the hypothesis that myocardial functional alterations associated with SH are dynamic and potentially reversible. Unlike our study, however, Catli et al. also reported differences in LV geometry at baseline. This discrepancy may be explained by differences in the study populations, as their cohort included children with both mild and severe SH (TSH 5–20 µIU/L) and heterogeneous etiologies, whereas our study was restricted to children with mild, idiopathic SH. Similarly, another study [60] involving children with severe SH of various etiologies, reported abnormalities in several indices of systolic and diastolic function, whereas IVRT remained within the normal range.

We acknowledge that our study has several limitations. First, the sample size was relatively small. Second, we were unable to compare the outcomes of treated SH children with those of an untreated SH cohort after two years of follow‑up; the inclusion of such a third arm would have enabled a clearer assessment of the specific effects of L‑T4 therapy on cardiovascular parameters in this population.

However, the strengths of this study include its prospective design and the selection of a homogeneous cohort of children with mild, idiopathic SH and a clearly defined duration of the condition. In particular, the absence of confounding underlying conditions allows for a better assessment of the direct effects of subclinical thyroid dysfunction and LT4 treatment on cardiovascular health. We acknowledge that the subtle cardiac alterations associated with SH may have greater clinical and prognostic relevance in patients with coexisting conditions than in otherwise healthy individuals. Accordingly, our findings should not be generalized to all pediatric patients with SH but rather interpreted as applicable to a carefully selected population without significant comorbidities.

Moreover, to the best of our knowledge, this is the first study to investigate the effects of long‑term (2‑year) L‑T4 supplementation on LV morphology and function in children with SH.

In conclusion, the results of this study provide evidence of subclinical early diastolic dysfunction without increased LV filling pressures in children with mild and persistent untreated idiopathic SH. IVRT emerges as the most sensitive marker of cardiac ultrasound-detectable modifications associated with untreated SH, whereas structural measures appear more variable in this population.

Notably, this functional alteration appears to be restored after two years of L‑T4 replacement therapy. Since it remains unclear whether this mild abnormality has any meaningful impact on daily activities, we cannot recommend routine cardiological evaluation for all children with persistent SH. Nevertheless, these patients should be monitored over time, and decisions regarding cardiovascular risk assessment and cardiac function evaluation should be made on an individual basis. Further randomized, controlled studies are needed to definitively establish the role of subclinical thyroid dysfunction on cardiovascular function and to determine the actual need for L-T4 treatment.

## Abbreviations

SH: Subclinical hypothyroidism
TSH: Thyroid-stimulating hormone
FT_4_: Free thyroxine
L-T_4_: L-thyroxine
Tg-Ab: Antithyroglobulin antibodies
TPO-Ab: Antithyroperoxidase antibodies
BMI: Body mass index
SDS: Standard deviation score
SBP, DBP: Systolic, diastolic blood pressure
CV: Cardiovascular
LV: Left ventricle
LVEF: Left ventricular ejection fraction
EDV, ESV: Left ventricular end-diastolic, end-systolic volume
RWT: Relative wall thickness
IVRT: Isovolumic relaxation time
MdT: Mitral deceleration time
TDI: Tissue Doppler imaging
